# Novel Bispecific Antibody for Synovial-Specific Target Delivery of Anti-TNF Therapy in Rheumatoid Arthritis

**DOI:** 10.3389/fimmu.2021.640070

**Published:** 2021-02-19

**Authors:** Mathieu Ferrari, Shimobi C. Onuoha, Liliane Fossati-Jimack, Alessandra Nerviani, Pedro L. Alves, Sara Pagani, Cecilia Deantonio, Federico Colombo, Claudio Santoro, Daniele Sblattero, Costantino Pitzalis

**Affiliations:** ^1^Department of Experimental Medicine and Rheumatology, Barts and the London School of Medicine and Dentistry, William Harvey Research Institute, Queen Mary University of London, London, United Kingdom; ^2^Department of Health Sciences and Interdisciplinary Research Center of Autoimmune Diseases, University of Eastern Piedmont, Novara, Italy; ^3^Department of Life Sciences, University of Trieste, Trieste, Italy

**Keywords:** rheumatoid arthritis, anti-TNF therapy, bispecific antibody, targeted therapy, biological drugs

## Abstract

Biologic drugs, especially anti-TNF, are considered as the gold standard therapy in rheumatoid arthritis. However, non-uniform efficacy, incidence of infections, and high costs are major concerns. Novel tissue-specific agents may overcome the current limitations of systemic administration, providing improved potency, and safety. We developed a bispecific antibody (BsAb), combining human arthritic joint targeting, *via* the synovial-specific single-chain variable fragment (scFv)-A7 antibody, and TNFα neutralization, *via* the scFv-anti-TNFα of adalimumab, with the binding/blocking capacity comparable to adalimumab -immunoglobulin G (IgG). Tissue-targeting capacity of the BsAb was confirmed on the human arthritic synovium *in vitro* and in a synovium xenograft Severe combined immune deficient (SCID) mouse model. Peak graft accumulation occurred at 48 h after injection with sustained levels over adalimumab-IgG for 7 days and increased therapeutic effect, efficiently decreasing tissue cellularity, and markers of inflammation with higher potency compared to the standard treatment. This study provides the first description of a BsAb capable of drug delivery, specifically to the disease tissue, and a strong evidence of improved therapeutic effect on the human arthritic synovium, with applications to other existing biologics.

## Introduction

The development of biologic agents for cancer and autoimmune disorders has revolutionized the standard therapeutic approach. Despite the undisputed success of several biologics currently in clinical use, some aspects are still a major source of concern. Systemic distribution and off-site on-target effects are still unsolved issues which can lead to the lack of potency and severe side effects in a subset of patients, constituting some of the principal drawbacks associated with this powerful class of therapeutics (1). Although the industry is in constant search for novel therapeutic targets to improve potency and overcome these limitations, an alternative approach could rely on improved tissue-specific delivery. Tailored drug delivery approaches could substantially reduce risks associated with the systemic exposure, improving safety and potency of new or established biological drugs. Rheumatoid arthritis (RA) represents an example of a severe chronic inflammatory condition localized mainly to an organ system (the joint) where tissue-specific therapeutic targeting could provide benefits to the patients.

Rheumatoid arthritis represents the most common and severe form of inflammatory arthritis with significant association with morbidity and mortality (2, 3). It affects ~1% of the adult population in Western Europe with an average age of onset of 40 years and an increase in incidence with rising age (4). The pathogenic processes in RA involve disequilibrium in the cytokine network in favor of pro-inflammatory stimuli, with elevated expression of key cytokines such as tumor necrosis factor (TNF), interleukin (IL)-1β, and IL-6 (5).

Strategies for RA therapy involve the use of non-steroidal anti-inflammatory drugs (NSAID) and synthetic disease-modifying antirheumatic drugs (sDMARD) as first-line treatments. Recent advances in the development of biologic DMARD (bDMARD) have opened the gates to the anti-cytokine era, leading to the rise of the anti-TNF biologics, currently considered the gold standard care for RA (6). However, a sizeable proportion of patients (30–40%) do not respond adequately, and treatment-free remission is still rarely achieved (6–8). It is plausible that different rates and efficiencies of tissue penetrance and accumulation, associated with suboptimal cytokine blockade at the site of interest, could explain anti-cytokine treatment resistance. Increasing drug concentrations in the disease tissues through a more tailored tissue-specific approach has the potential to improve the therapeutic range without increasing the systemic dose and the associated risk of toxicity.

Bispecific antibodies (BsAbs) are gaining momentum with increasing clinical success, as an emerging class of biological therapeutics characterized by simultaneous binding capacity to two distinct epitopes. This has been successfully applied in cancer therapy, with bispecific constructs being able to interact with CD3 and cancer-specific antigens to activate effector cells in the proximity of the disease tissues (9). Here, we describe a bispecific construct for the treatment of RA by combining a well-established anti-TNF therapeutic domain [single-chain variable fragment (scFv)-adalimumab, Humira™, AbbVie Inc. North Chicago, IL, USA] with a tissue-targeting domain we previously described (scFv-A7) (10). The scFv-A7 showed remarkable tissue and disease specificity for the microvascular compartment of the human arthritic synovium, with no detectable reactivity with a vast array of human tissues, including normal human synovium and other inflammatory diseases (10). ScFv-A7 antibody displays all properties of an ideal candidate for targeted therapy in RA, with expectations of increasing therapeutically relevant concentrations within the disease tissue while reducing systemic distribution. The results reported here represent the first preclinical evidence of tissue-specific delivery and therapeutic efficacy within the disease tissues using a commonly prescribed bDMARD that may pave the way to translating this approach into patients.

## Materials and Methods

### Antibody Cloning Strategy

ScFv-A7 VH/VL sequences were obtained from the phage display clone (10). E2 (anti-dipeptidyl aminopeptidase-like protein 6) scFv-Fc sequence was kindly provided by Professor D. Sblattero. adalimumab sequence was obtained from the patent (11). All adalimumab were cloned in fusion with the IgG_1_ Fc CH2–CH3 region, and bispecific constructs in pDuo vectors, including the Y407T (for adalimumab) or the T366Y mutation (for A7 and E2) (12, 13).

### Antibody Expression and Characterization

Antibodies were expressed by stably transfected Chinese hamster ovary (CHO)-S cell line and purified using Talon® Metal Affinity Resins (Clontech Laboratories, Inc., Mountain View, CA, USA), for BsAb A7/adalimumab and E2/adalimumab, or protein A Sepharose CL-48 resin (GE Healthcare, Chicago, IL, USA) for A7 scFv-Fc and adalimumab scFv-Fc. Antibodies were biotinylated using an EZ-Link Sulfo-NHS-SS Biotinylation Kit (ThermoFisher Scientific Inc., Waltham, MA, USA) according to the manufacturer's protocol. Antibody purity was determined by SDS-PAGE *via* SYPRO Ruby Protein Gel Stain (Life Technologies, Carlsbad, CA, United States).

### Human Tissues

Synovia from patients with RA were obtained during joint replacement surgery, and skin tissues were obtained from patients undergoing plastic surgery. All patients had given informed consent as approved by the institutional ethics committee (REC 05/Q0703/198). Samples were frozen using CryoStor® (Sigme-Aldrich, St. Louis, Mo, USA) freeze media according to the manufacturer's instructions and stored at −80°C until usage.

### Animals

Our own colony of the SCID mice (SCID beige mouse, CB17.Cg-PrkdcSCIDLyst^bg−J^/Crl mice) were housed at Charles River Laboratories and handled at Queen Mary University of London during the experimental procedure in accordance with the institutional guidelines and procedures approved by the UK Home (Scientific Procedures Act 1986, Project License PPL 7008259).

### Determination of Anti-TNF Activity

Anti-TNF ELISA, cytotoxicity assay, and surface plasmon resonance (SPR) analysis were performed as previously described (14).

### Histology

Analysis of tissue morphology on frozen tissue sections was performed upon acetone fixation by H&E staining on six sections per sample spanning 120 μm. Immunohistochemistry on frozen tissue sections were stained with mouse anti-human CD3, CD20, CD68, CD90 or von Willebrand factor (vWF), and CD31 (Dako, Hamburg, Germany), according to the manufacturer's recommendations, and detected with EnVision™ horseradish peroxidase (HRP; Dako) and a 3,3′-diaminobenzidine (DAB; Dako) substrate solution. Staining was performed on four sections per sample spanning 120 μm. Immunofluorescence on frozen tissues were stained with 20 μg/ml of biotinylated test antibody and mouse anti-human vWF (Dako) at 1 μg/ml. Biotinylated antibodies were detected with streptavidin-conjugated Alexa Fluor 488 (Life Technology), and anti-vWF was detected with a goat anti-mouse IgG Alexa Fluor 555 (Life Technology). Immunohistochemistry on formalin-fixed paraffin-embedded tissue sections were dewaxed, and the antigen was retrieved following proteinase K digestion (Dako). Sections were incubated with 20 μg/ml of biotinylated antibody and detected with streptavidin-HRP, followed by DAB incubation. Quantification of tissue cellularity from H&E and immunohistochemistry was performed using cellSens Dimension (Olympus, Tokyo, Japan) *via* automated cell count using adaptive thresholding. Cellular density was expressed as % of the tissue area covered by pixels above threshold.

### Synovium Xenograft SCID Mouse Model

Rheumatoid arthritis synovium and skin tissues were thawed from liquid nitrogen immediately before surgery, washed, and then kept in RPMI 1640 medium. Beige male and female SCID mice (6–8 weeks old) were housed in groups of six per cage. The mice were anesthetized, and biopsies were inserted subcutaneously in the dorsal skin (two synovium biopsies over the scapulae, two skin biopsies over the kidney region), as previously described (15). To avoid the cage effect, mice were randomized into treatment groups within a cage immediately before the first injection. Mice were injected intravenously with 3 mg/kg of test antibodies at day 7 and 14 post-transplant. The sample size was calculated according to the previous studies (16, 17). The operator performing the experiment was blind for the treatment. Animals were culled 21 days post-transplant, tissues were harvested for RNA extraction and histology, and blood were collected for cytokine measurements (some of the grafts have been excluded due to the limited amount of tissue). Six mice per group were used for a total of 12 synovium and 12 skin grafts.

### *In vivo* Imaging

*In vivo* fluorescence images at various time points after intravenous (IV) injection of 3 mg/kg Cy5.5 labeled Ab (6, 24, 48, 72, 144, and 168 h) were acquired and analyzed with a Lumina II™ IVIS (*In Vivo* Imaging System) instrument (Caliper Life Sciences, Waltham, MA, USA) with quantification of bioluminescence performed using Living Image™ software (Caliper Life Sciences) according to the manufacturer's instructions. Mice were maintained under isoflurane inhalation anesthesia. Control animals were injected with Phosphate-buffered saline (PBS). The signal emitted by grafts Region of interest (ROI) was measured, and data expressed as radiant efficiency and quantified as photons s^−1^ cm^−2^ sr^−1^.

### RNA Expression

The RNA from grafts was extracted using TRIZOL as previously described (16) and quality checked at the NanoDrop 2000C (Labtech, Heathfield, UK). A maximum of 500 ng total RNA was reverse transcribed and the generated cDNA stored at −20°C until use.

Gene expression profiling was performed by quantitative real-time PCR using 15 ng/well of cDNA in an ABI Prism 7900HT Fast Real-Time PCR System (Applied Biosystems, Foster City, CA, USA). Relative expression was calculated using the comparative threshold cycle (Ct) method; Ct cycle of the human endogenous control glyceraldehyde-3-phosphate dehydrogenase (GAPDH) was used to normalize for the initial cDNA amount. Samples with Ct value of GAPDH ≥ 26 were excluded from the analysis.

### Cytokine Analysis in SCID Mice Sera

Mice sera were collected through terminal bleeding at day 14. Quantification of human cytokines concentrations was performed using human-specific V-Plex immunoassay (Meso Scale Discovery, Rockville, MA, USA) and analyzed using a QuickPlex SQ 120 instrument (Meso Scale Discovery) according to the manufacturer's recommendations.

### Statistical Analysis

All data sets were first screened with the ROUT test to identify statistically significant outliers (18). Statistical comparison within treatment groups was performed using the Mann–Whitney unpaired non-parametric test in GraphPad Prism v6 (GraphPad Software Inc., San Diego, CA, USA).

## Results

### Bispecific Antibody Design

**Figure 1:**
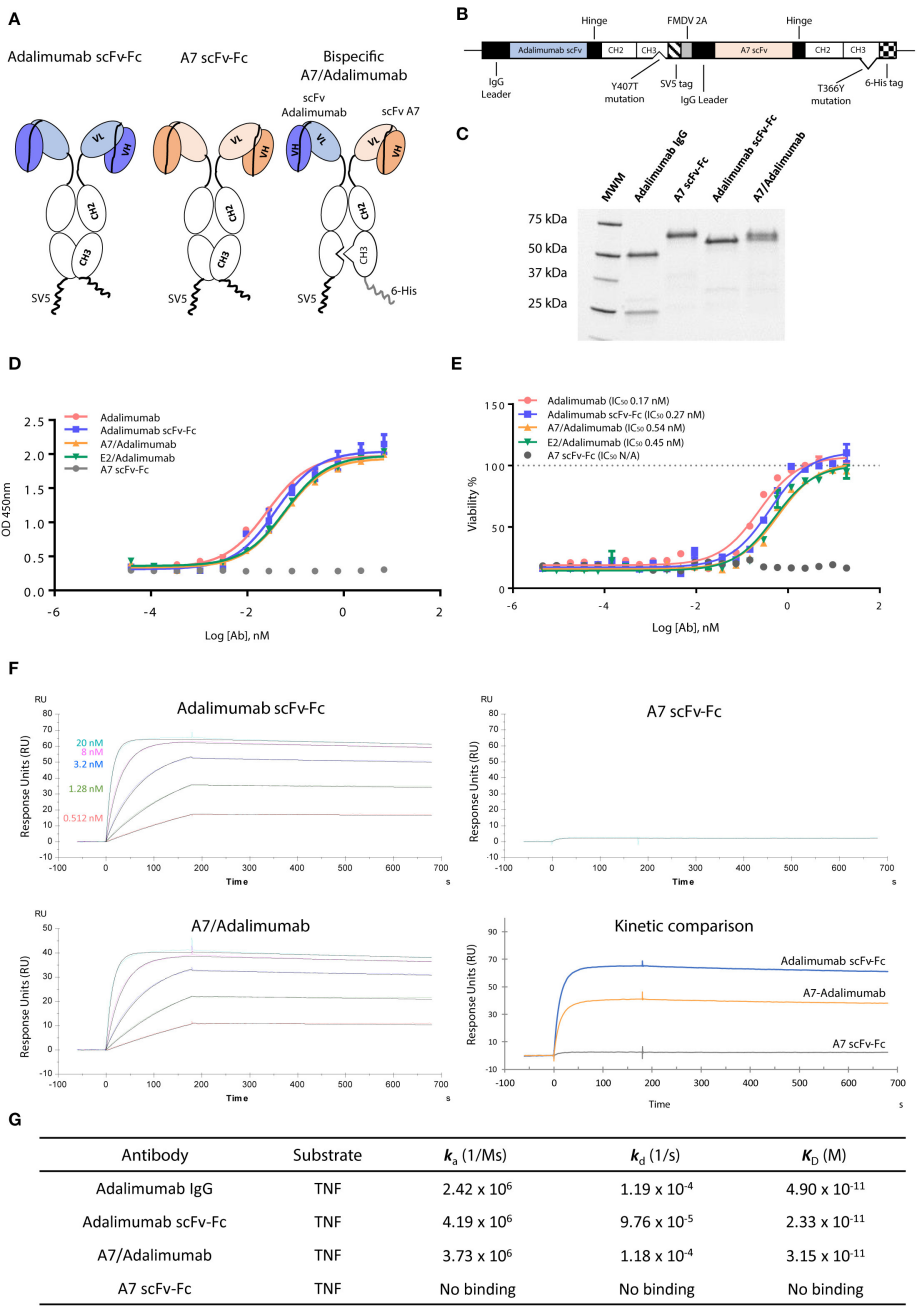
Characterization of scFv-Fc antibody constructs. **(A)** ScFv of interest was cloned in fusion with the immunoglobulin (IgG) Fc region as single-chain variable fragment (scFv)-Fc construct. Bispecific scFv-Fc antibody carried the A7 scFv-Fc fused to the Fc chain containing the “hole” T366Y mutation while the adalimumab scFv-Fc chain contained the “knob” Y407T substitution. **(B)** Bispecific antibody expression vector contained the adalimumab scFv sequence fused to the CH2-CH3 (Y407T) Fc domain and the SV5 tag while the A7 scFv was fused to the CH2-CH3 (T366Y) Fc region and to a 6-His tag. The two chains were linked in a monocistronic sequence via the FMDV 2A peptide to allow an equal expression of the two chains from a single RNA. **(C)** SDS-PAGE analysis of the scFv-Fc and bispecific scFv-Fc antibodies shows the presence of a single band around 60 kDa compared to the heavy (50 kDa) and light (25 kDa) chains visible for the parent adalimumab IgG antibody. **(D)** Tumor necrosis factor (TNF) binding capacity of bispecific antibodies and reference adalimumab IgG and scFv-Fc antibodies as determined *via* ELISA. Both bispecific antibodies and adalimumab scFv-Fc format showed comparable TNF binding capacity to the parent adalimumab IgG. No binding could be detected with the A7 scFv-Fc antibody. **(E)** Neutralization of TNF in a TNF induced cytotoxicity assay using L-929 cell line was efficiently obtained with the bispecific antibodies as expressed by the 50% inhibitory concentration (IC_50_). Potencies similar to adalimumab IgG and adalimumab scFv-Fc were determined for both bispecific antibody constructs. No rescue of cellular viability was obtained with the A7 scFv-Fc antibody. Values expressed as mean ± SEM. **(F)** Surface plasmon resonance sensorgrams representing binding kinetics of adalimumab scFv-Fc and A7/adalimumab to TNF. TNF concentrations were 20 nM (light blue), 8 nM (purple), 3.2 nM (blue), 1.28 nM (green), and 0.512 nM (red). Kinetic comparison between adalimumab scFv-Fc, A7-adalimumab, and A7 scFv-Fc is expressed with the sensorgram of interaction with TNF at 20 nM. **(G)** Surface plasmon resonance kinetic measurements of anti-TNF antibodies. *ka*, association rate constant; *kd*, dissociation rate constant; KD, affinity constant. Adalimumab IgG kinetic measurements were previously reported in the literature (14).

To obtain maximum therapeutic potency, the anti-TNF antibody adalimumab was selected as the effector moiety. The parent adalimumab IgG antibody was first converted in the scFv format, supported by the scFv origins of adalimumab (19), maintaining binding specificity and pharmacokinetics as previously reported (20, 21). As tissue targeting partner, the scFv-A7 was selected for its arthritic synovium specificity (10). All antibodies were demonstrated to be in the expected format when expressed as scFv-Fc fusion constructs (Figure 1).

To create a BsAb, we adopted the “knob-into-hole” technology based on the replacement of a small amino acid with a larger one (T366Y) in the IgG CH3 domain of one chain and an opposite substitution in the second chain (Y407T), preventing homodimerization due to steric hindrance and protein instability (22). To avoid issues related to heavy and light chain mispairing, common in most of the IgG-like BsAb formats, the variable domains were designed as scFv (Figure 1A). To ensure a correct 1:1 ratio of expression and increase heterodimerization efficiency, the two chains were coded in the same vector linked *via* the 2A self-processing peptide as previously described (12), further processed by a furin cleavable site to ensure complete removal of the 2A peptide sequence (Figure 1B). The BsAb scFv-Fc format for A7/adalimumab is therefore a dimer containing both variable domains and the Fc IgG region with an approximate molecular weight of 120 kDaadalimumab, resulting in two bands at 60 kDa in a reduced SDS-PAGE, corresponding to the A7 scFv-Fc and the adalimumab scFv-Fc chains, confirming heterodimer formation (Figure 1C). All three antibodies showed comparable stability as determined by differential scanning fluorimetry (DSF) (Supplementary Figure 1). Additionally, a high degree of heterodimerization of our system was confirmed using a truncated scFv-Fc bispecific construct as described in Supplementary Figure 2.

A non-targeting bispecific construct comprising the scFv-E2 and scFv-adalimumab (E2/adalimumab) was also generated as the negative control.

### Characterization of *in vitro* Targeting and Therapeutic Function

TNF binding and neutralization were investigated using an ELISA and a cytotoxic assay. Despite the conversion to scFv format, the adalimumab scFv-Fc antibody showed almost identical binding capacity for TNF when compared to the parent adalimumab IgG antibody. A7/adalimumab and the control of E2/adalimumab bispecific antibodies displayed TNF binding capacity with a slight half-maximal effective concentration (EC_50_) shift (Figure 1D). TNF-induced cytotoxicity assay showed a similar neutralizing dose–response curve between adalimumab IgG and adalimumab scFv-Fc antibodies, while no rescue of cellular viability was detected with A7 scFv-Fc. Comparable TNF inhibition was also determined for the bispecific antibodies with a half-maximal inhibitory concentration (IC_50_) characterized by a 2-fold increase compared to adalimumab scFv-Fc (Figure 1E). This increase in EC_50_ and IC_50_ is most likely due to the presence of only one anti-TNF domain and consequently a halved TNF binding/blocking potential.

TNF binding kinetics and association (*k*_a_) and dissociation (*k*_d_) rates were further characterized *via* SPR (Figures 1F,G). The previously published data on adalimumab IgG (14) shows comparable kinetics to the scFv-Fc format and to the bispecific A7/adalimumab antibody with an affinity (*K*_D_) of 49, 23, and 33 pM, respectively, further demonstrating unaltered TNF interaction profiles for the newly developed antibody formats. No TNF binding was detected with the A7 scFv-Fc antibody. Interestingly, while the kinetics remained unchanged, the maximal response of the A7/adalimumab BsAb at saturating concentrations of TNF (20 nM) was only half of what was detected for adalimumab scFv-Fc (Figure 1F). These data confirm that the monovalent TNF binding of the bispecific format does not affect the kinetics of interaction with soluble TNF but only results in a reduced number of interactions. It is also apparent that one paratope on a single arm can efficiently target and block the TNF trimer, further supporting the development of bispecific antibodies with only one anti-TNF domain.

**Figure 2:**
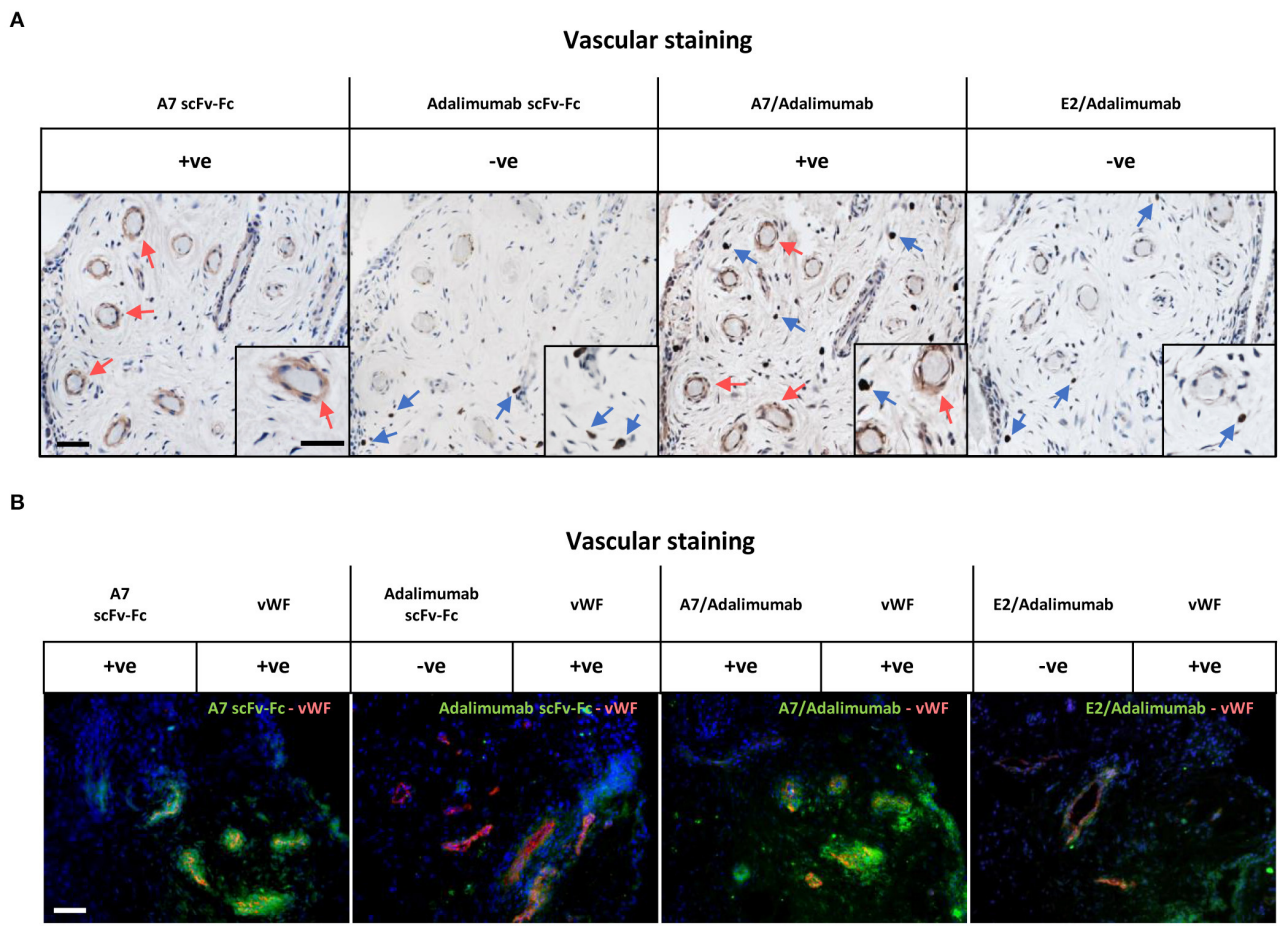
Reactivity in the human arthritic synovium. **(A)** Immunohistochemistry on human arthritic synovium stained with biotinylated A7 scFv-Fc, adalimumab scFv-Fc, A7/adalimumab and E2/adalimumab at 20 μg/ml (*n* = 3). Binding of antibody to tissue section detected with horseradish peroxidase (HRP) conjugated avidin–biotin complex. Red arrows indicate A7 staining. Blue arrows indicate anti-TNF staining. Insert represents magnification of vasculature and reactive cells. **(B)** Immunofluorescent staining on arthritic synovium with biotinylated A7 scFv-Fc, adalimumab scFv-Fc, bispecific A7/adalimumab, and bispecific E2/adalimumab at 20 μg/ml in combination with anti-von Willebrand factor (vWF). Biotinylated antibody detected with streptavidin-Alexa Fluor 488 (green). Anti-vWF detected with anti-mouse Alexa Fluor 555 (red). DAPI counterstain to depict nuclei (blue). Scale bar in representative pictures 100 μm.

We then tested the capacity of the BsAb A7/adalimumab to retain tissue targeting capacity using immunohistochemistry (Figure 2A) and immunofluorescence (Figure 2B) in the human arthritic synovium (*n* = 3) as previously described for scFv-A7 (10). Strong reactivity was detected with the perivascular compartment of the synovial microvasculature with a similar pattern to the reference A7 scFv-Fc antibody. No staining could be detected with the E2/adalimumab BsAb, confirming its suitability as a non-targeting control antibody.

### *In vivo* Localization of A7/Adalimumab BsAb in the Human Synovium-SCID Mouse Xenograft Model

The tissue localization properties of the BsAb A7/adalimumab to the human arthritic synovium were addressed by using a Cy5.5 conjugated construct injected intravenously into SCID mice double grafted with the human arthritic synovium (*n* = 4) and control human skin (*n* = 4). The use of this xenograft mouse model of arthritis provides a unique opportunity to evaluate therapeutic accumulation in human tissues. Further, the presence of the normal human skin graft serves as an internal control for non-targeted tissue accumulation.

**Figure 3:**
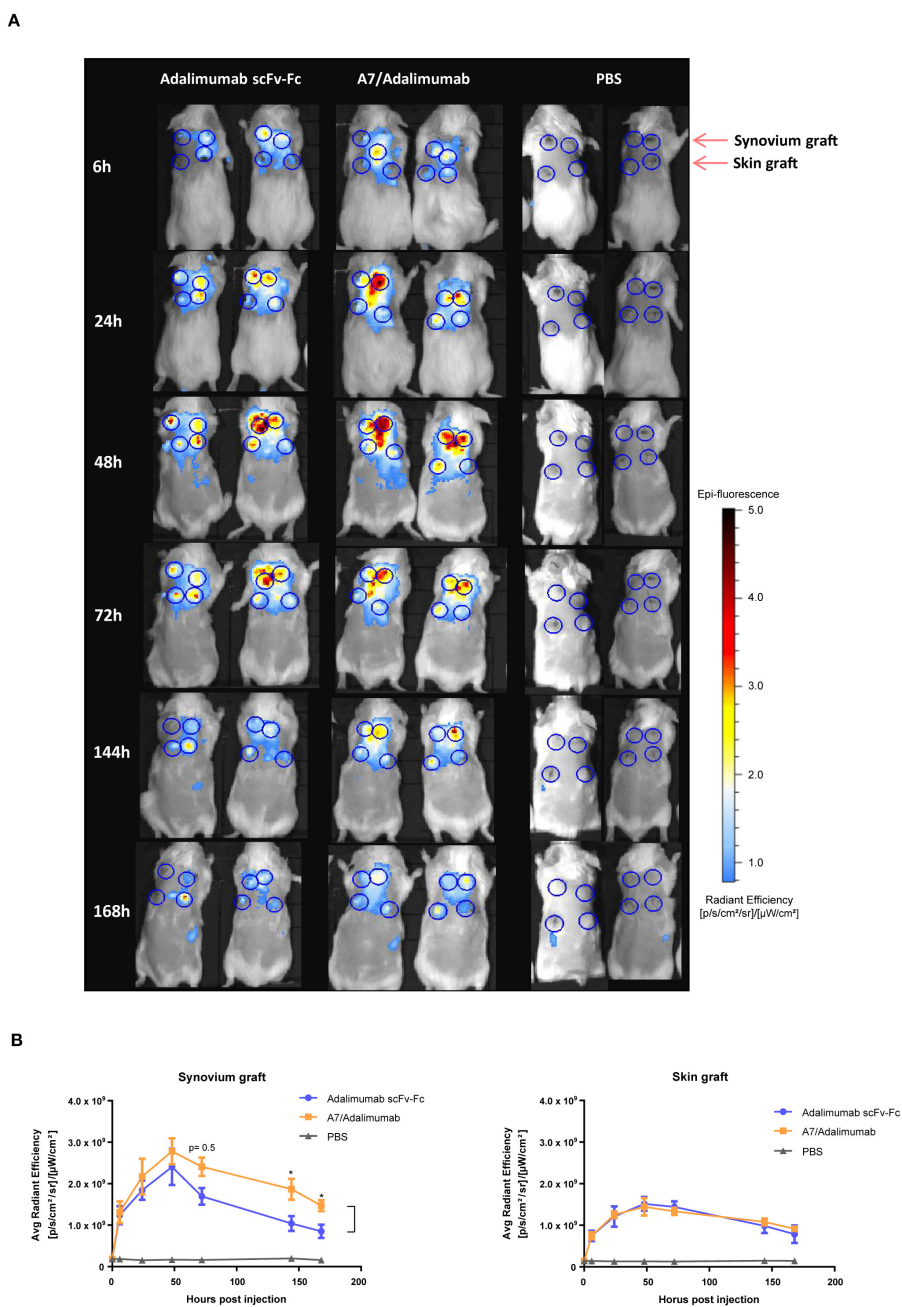
*In vivo* localization in human synovium xenograft SCID mouse model. **(A)** SCID mice double grafted with the human arthritic synovium (top) (*n* = 4) and human normal skin (bottom) (*n* = 4) were injected intravenously with Cy5.5 conjugated antibody and imaged at 6, 24, 48, 72, 144, and 168 h. As control, PBS was injected. Blue circles indicate graft location in fluorescence images. **(B)** Quantification of average radiant efficiency in the region of interest encompassing the graft location shows an increased intensity that correlates with the amount of Cy5.5 conjugated antibody accumulated in the tissue. Values were expressed as mean ± SEM. Mann–Whitney non-parametric test (*p < 0.05).

Antibody localization in the grafts was assessed *via* fluorescent imaging starting at 6 h post-injection and 24 h increments until day 7. *In vivo* fluorescent imaging showed a preferential synovial accumulation for adalimumab scFv-Fc and A7/adalimumab BsAb starting at 24 h with a signal detectable up to 7 days post-injection (Figure 3A). Analysis of the average radiant efficiency in the graft region revealed that the peak of accumulation of A7/adalimumab in the synovial graft occurred at 48 h with significantly prolonged retention in the target tissue compared to adalimumab scFv-Fc for up to 7 days (*p* < 0.05) (Figure 3B). Further, in the human skin graft used as negative control, both antibodies demonstrated similar basal levels of fluorescence (Figure 3B). Comparison of the average radiance efficiency between synovium grafts and skin grafts revealed a significantly higher synovial tissue accumulation for the BsAb A7/adalimumab between 48 h and 7 days post-injection (*p* < 0.05) while adalimumab scFv-Fc, although not statistically significant, exhibited a higher fluorescence intensity in the synovial grafts only at 48 h (*p* = 0.1) that quickly resolved to the levels measured for the skin grafts at 72 h post-injection (Figure 3B, Supplementary Figure 3). Further, human IgG serum levels 1 week after injections were significantly higher in the A7/adalimumab treatment group, indicating an increased half-life (Supplementary Figure 4).

### Therapeutic Efficacy of A7/Adalimumab in the Human Synovium-SCID Mouse Xenograft Model

In order to establish that BsAb A7/adalimumab maintained therapeutic efficacy *in vivo*, we adopted the human synovium-SCID mouse xenograft model. The mice were treated 1 week after grafting with IV injections at day 0 and 7 of 3 mg/kg adalimumab scFv-Fc, A7/adalimumab, E2/adalimumab (non-targeting antibody), or A7 scFv-Fc and PBS (control group). Six animals per group were receiving two human skin and two arthritic synovium grafts (*n* = 12). Grafts were harvested at day 14 and divided for immunohistochemical analysis to measure cellularity levels and mRNA extraction for molecular profiling. Tissues with satisfactory human GAPHD mRNA expression levels (≤26 PCR amplification cycles) were considered viable and retained for further analysis.

**Figure 4:**
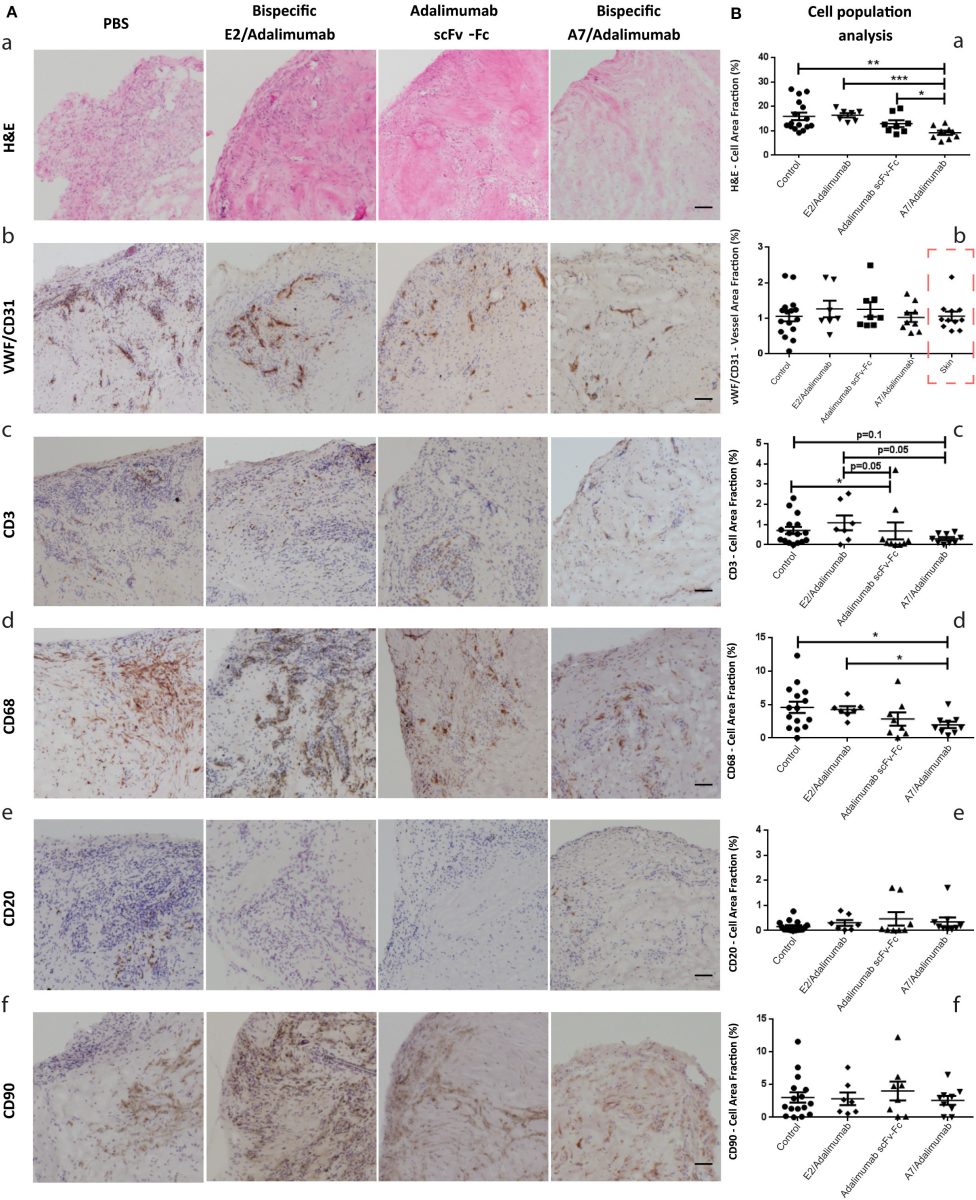
Histomorphological features of human synovium xenografts. **(A)** Immunohistochemical analysis of synovial tissues from xenograft mice receiving PBS or A7 scFv-Fc (control group), adalimumab scFv-Fc, A7/adalimumab, or E2/adalimumab. H&E staining reveals the total cell population within the tissues, displaying a reduced cellularity in the A7/adalimumab group (a). Staining for specific cell types was performed with antibodies targeting vWF/CD31 (endothelial cells) (b), CD3 (T-cells) (c), CD68 (macrophages) (d), CD20 (B-cells) (e), and CD90 (fibroblasts) (f). Brown staining indicates the presence of antibody binding. The staining shows a reduced CD68 content in the A7/adalimumab and adalimumab scFv-Fc groups compared to control or non-targeting antibody E2/adalimumab. Similar vascular content, CD3^+^, CD20^+^, and CD90^+^ cells were present in all tissues. **(B)** Stainings of graft tissue sections were analyzed with cellSens P imaging software tool using adaptive thresholding to quantify the area fraction of the stainings. H&E stainings were analyzed for hematoxylin stained nuclei content (a). vWF-CD31 staining was analyzed to quantify total vascular density across the treatment groups and synovium/skin grafts (red square to indicate values measured on skin xenografts) (b). CD68 (d), CD3 (c), CD20 (e), and CD90 (f) analyses revealed the cell area fraction for macrophages, T cells, B cells, and fibroblast, respectively. Control *n* = 16; adalimumab scFv-Fc *n* = 8; A7/adalimumab *n* = 9; E2/adalimumab *n* = 7. Values were expressed as mean ± SEM. Mann–Whitney non-parametric test (***p < 0.001, **p < 0.01, *p < 0.05).

As shown in Figure 4Aa, varying degree of cellularity could be detected between the four treatment groups with consistently lower levels in the A7/adalimumab treated mice. A slight decrease in total cell content could also be detected in the adalimumab scFv-Fc group. The stained sections were analyzed *via* the imaging software to quantify the number of cells present per tissue section, revealing a significant reduction in the total cell content in the A7/adalimumab BsAb treatment compared to control, adalimumab scFv-Fc and E2/adalimumab (*p* < 0.01, *p* < 0.05, and *p* < 0.001, respectively) (Figure 4Ba). As the degree of tissue vascularization can affect graft survival and cell viability, tissue sections were stained for human vWF and CD31 to highlight the vasculature (Figure 4Ab). A similar vascular density was determined across all treatment groups and between synovium and skin grafts (Figure 4Bb).

Synovium grafts were further stained for CD3, CD68, CD20, and CD90 to identify specific cell populations (Figure 4Ac–f, respectively). A similar B-cell (CD20) and fibroblast (CD90) content was present in all tissues tested (Figure 4Be,f), while a decreasing trend for T cells (CD3) could be detected in the adalimumab scFv-Fc (*p* < 0.05 and *p* = 0.05) and A7/adalimumab (*p* = 0.1 and *p* = 0.05) treatment groups compared to control and E2/adalimumab (Figure 4Bc). Remarkably, the A7/adalimumab treatment was more effective in decreasing the number of CD68^+^ cells (macrophages) with levels significantly lower than control and E2/adalimumab groups (*p* < 0.05) (Figure 4Bd).

**Figure 5:**
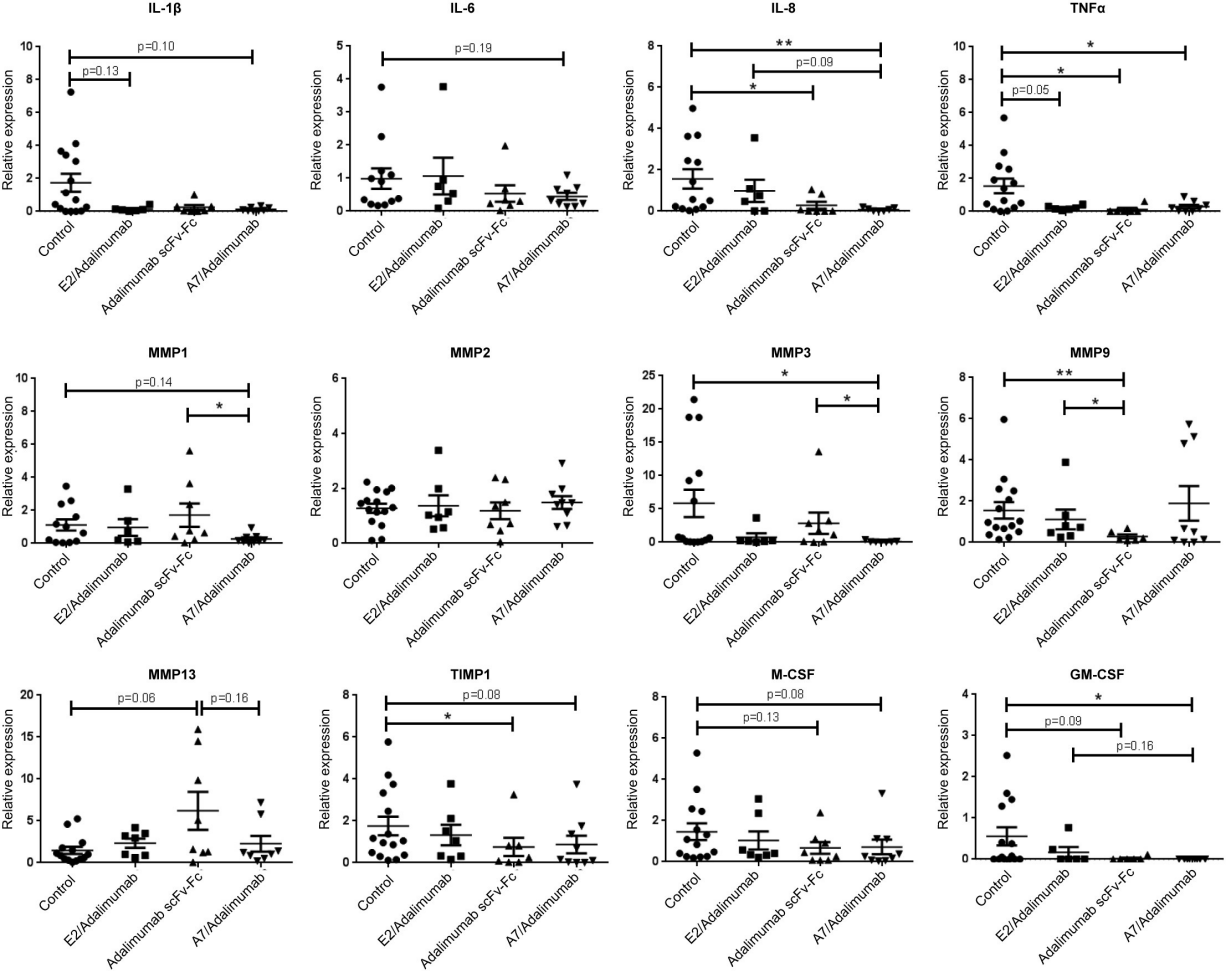
mRNA expression profiling in human synovium xenografts. mRNA expression in synovium grafts from mice receiving PBS and A7 scFv-Fc (control), adalimumab scFv-Fc, A7/adalimumab, or E2/adalimumab were represented as relative expression compared to the median of the control group. Grafts were harvested 7 days after second injection. Control *n* = 16; adalimumab scFv-Fc *n* = 8; A7/adalimumab *n* = 9; E2/adalimumab *n* = 7. Values were expressed as mean ± SEM. Mann–Whitney non-parametric test (*p < 0.05, **p < 0.01).

Molecular profiling for specific markers of inflammation was performed on synovial grafts from the four treatment groups to identify changes in molecular signatures of treatment response. The A7/adalimumab treatment group showed a marked decrease in pro-inflammatory cytokine mRNA expression. Levels of IL-8 and TNF were consistently lower than the control group (*p* < 0.01 and *p* < 0.05, respectively), while IL-1β and IL-6 levels showed a decreasing trend, without reaching statistical significance (*p* = 0.1) (Figure 5). These data indicate efficient TNF blockade with consequent downregulation of key cytokines as previously described (23, 24). The pro-inflammatory environment in RA synovium can stimulate synoviocytes of the lining layer to produce matrix metalloproteinases (MMP) (25–28). The expression of MMP-1,−2,−3,−9, and−13 mRNA was compared between the treatment groups. BsAb A7/adalimumab treatment was able to significantly reduce MMP-1 and MMP-3 mRNA expression compared to adalimumab scFv-Fc (*p* < 0.05). Levels of MMP-2,−9, and−13 did not show modulation compared to the control group, while adalimumab scFv-Fc was able to significantly reduce MMP-9 mRNA expression (*p* < 0.01) (Figure 5). Also, the levels of tissue inhibitor of metalloproteinases (TIMP)-1 mRNA were affected by adalimumab scFv-Fc treatment compared to control (*p* < 0.05) (Figure 5). Consistent with data already described in Figure 5, A7/adalimumab treatment resulted in a marked decrease of granulocyte-macrophage (GM)-CSF mRNAs compared to control (*p* < 0.05) (Figure 5).

**Figure 6:**
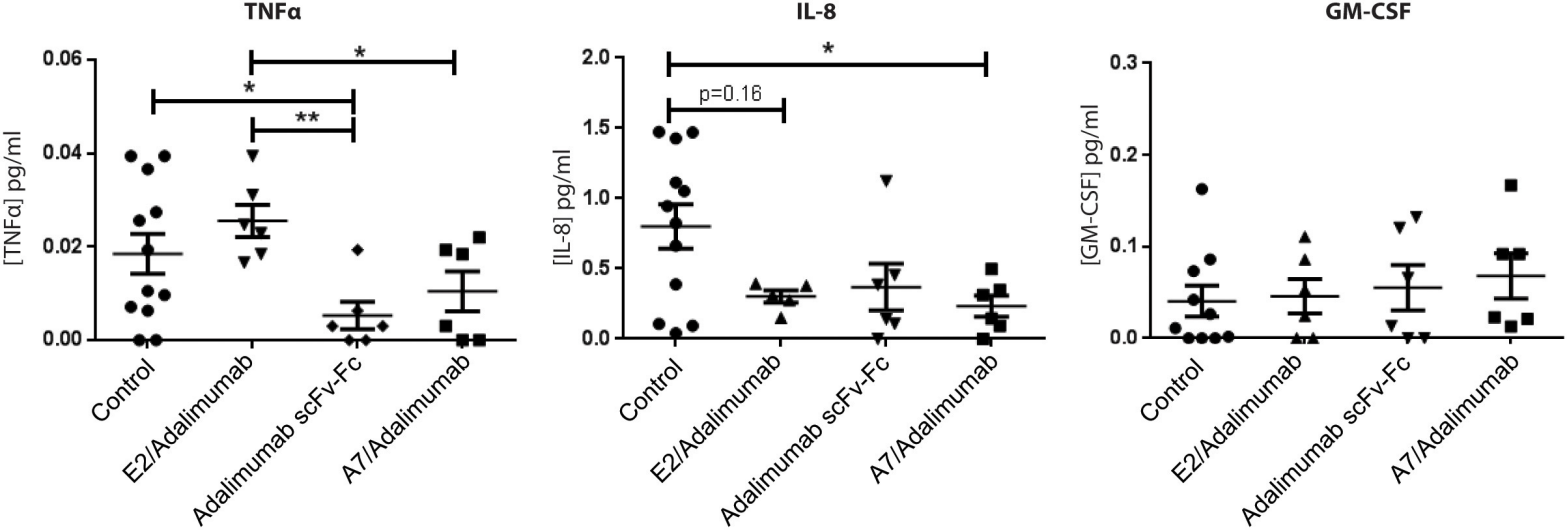
Human cytokine concentrations in mouse sera. Selected human cytokine concentrations in sera collected at day 14 from mice receiving PBS and A7 scFv-Fc (control) (*n* = 12), adalimumab scFv-Fc, A7/adalimumab, or E2/adalimumab (*n* = 6) were measured using multiplex sandwich immunoassay. Values were expressed as mean ± SEM. Mann–Whitney non-parametric test (**p < 0.01, *p < 0.05).

To confirm the reduced expression of pro-inflammatory markers detected in mRNA analysis, human cytokine concentrations in SCID mice sera were determined using a multiplex sandwich ELISA assay. Circulating levels of granulocyte-macrophage colony stimulating factor (GM-CSF) did not show significant changes across the treatments, while IL-8 serum concentrations were lower in the A7/adalimumab treatment group compared to control (*p* < 0.05). Interestingly, both adalimumab scFv-Fc and A7/adalimumab showed a marked decrease in soluble TNF levels compared to control and E2/adalimumab groups (*p* < 0.01 for adalimumab scFv-Fc and *p* < 0.05 for A7/adalimumab) (Figure 6). Taken together, these results indicate an efficient anti-TNF activity of the BsAb A7/adalimumab, resulting in a more potent anti-inflammatory effect compared to the parent Anti-TNF antibody.

## Discussion

Current successful therapeutics for the treatment of RA are based on blockade of pro-inflammatory pathways, targeting cytokines or immune effector cells. Although clinically efficacious due to the crucial importance of these pathways in immune surveillance/response, their targeting can often lead to increased risk of infections and/or reactivation of latent diseases such as tuberculosis (6, 29, 30). Furthermore, the systemic broad distribution of these targets may reduce the effective therapeutic dose reaching successfully the tissue of interest. This will in turn require an increased administration dose, with the associated risk of toxicity, to reach the optimal therapeutic concentration range in the disease tissue (23, 31). The development of a therapeutic molecule containing a second moiety with the tissue and/or disease specificity represents a powerful approach to increase pharmaceutical potency and reduce systemic side effects. Antibodies are exceptionally suited for this task due to their binding specificity and versatility, embodying the “magic bullet” concept conceived by Paul Ehrlich (32).

Several constructs are currently in development for the treatment of rheumatoid arthritis in the preclinical or early clinical stage as have been extensively reviewed (1). An example of this is the development of scFv antibody fragments against extra-domain (ED) A and B splicing variants of fibronectin that are reexpressed during inflammation and cancer and have been successfully conjugated with effector molecules (33–36). Our group and collaborators demonstrated the successful delivery of IL-4 *in vivo* using a synovium targeting peptide in the xenograft SCID mouse model, and the selective accumulation of an anti-C5 antibody fused to the same synovium targeting peptide in affected joints of rodent models of arthritis (37, 38). Similarly, the synovium targeting peptide conjugated with methotrexate-loaded nanoparticles increased the accumulation of these nanostructures in inflamed joints (animal models of arthritis) compared to naked nanoparticles and showed higher efficacy in comparison with conventional doses of methotrexate (39), further confirming the validity of targeted therapy in an arthritic context.

The scFv antibody adopted in this study (scFv-A7) proved to specifically target the microvascular compartment of the human arthritic synovium with no detectable reactivity with other human tissues, both in healthy and disease conditions (10). This elevates it as a unique tool for the treatment of the human arthritic condition, with increased disease tissue specificity, and consequently improved safety, compared to more widespread moieties such as the anti-extradomain A of fibronectin (anti-EDA) or similar inflammation targeting domains.

Ligand-based vascular targeting of disease tissue is an attractive strategy for the treatment of conditions characterized by extensive neo-angiogenesis and vascular remodeling. This approach is likely to improve the therapeutic index of available therapeutic agents, increasing safety by targeting disease-specific antigens in the vascular compartment and selectively delivering drug *in vivo*, sparing healthy organs. These features underpinned our strategy of combining the gold standard for anti-TNF therapy adalimumab (6) with scFv-A7 for functional tissue-specific delivery in a BsAb format, providing proof of concept in RA. The use of an scFv-Fc fusion structure is known to prevent heavy and light chain mispairing and contribute to increasing heterodimerization efficiency. In addition, this format has been shown to maintain long serum half-life and secondary Fc effector functions, such as antibody-dependent cellular cytotoxicity and complement-dependent cytotoxicity (12, 22). Adalimumab was shown to maintain binding and neutralizing capacity as an scFv compared to the IgG format (19, 21), which was preserved in the BsAb design described here. The scFv-Fc format of adalimumab showed TNF binding capacity with similar kinetic properties compared to the original antibody (14). In addition, the scFv-Fc antibody was biologically active, rescuing L-929 cells from TNF-induced cytotoxicity with an IC_50_ value closely comparable to the parent antibody. Despite adalimumab having been shown to form complex multimeric structures with soluble TNF, with the most stable binding configuration consisting of three separate TNF homotrimers and three adalimumab antibodies (40), the monovalent nature of the A7/adalimumab BsAb construct was still able to bind TNF with comparable kinetics and to efficiently block TNF biological activity.

Importantly, the BsAb A7/adalimumab proved to be more effective than the adalimumab scFv-Fc antibody in localizing the target tissue *in vivo*, maintaining significantly higher levels over the course of 7 days with a single injection. Due to the presence of higher TNF content in the synovial graft compared to the control human skin graft, it is plausible to speculate that the initial accumulation of adalimumab scFv-Fc at 48 h may be driven by the presence of TNF in the target tissue. However, a rapid decrease to the levels observed in the skin graft was determined only for adalimumab scFv-Fc, while the BsAb A7/adalimumab retained levels above both skin graft and parent antibody for the duration of the treatment. Analysis of synovium and skin grafts revealed a comparable vascular density, ruling out the effect of degree of vascularization as the driving force for synovial accumulation. Further, the previous data on this model have shown that an injected non-targeting isotype control antibody would still show synovial grafts uptake, reminiscent of our adalimumab scFv-Fc (17). As all the constructs described here are characterized by a similar structure and molecular weight, passive tissue diffusion and serum half-life in SCID mice are expected to be comparable. This suggests that the A7-targeting domain is responsible for faster tissue localization and sustained intra-graft concentration over what can be expected solely by passive distribution.

Therapeutic efficacy was evaluated using the human synovium xenograft SCID mouse model, as opposed to the classic collagen-induced arthritis (CIA) model. Although the xenograft model does not allow the evaluation of cartilage and bone erosions associated with RA, it does offer the unparalleled advantage of directly testing the effect of human-specific biologic agents, such as the anti-TNF adalimumab, on cellular infiltration and cytokine production in its natural human microenvironment, accelerating translation to the human disease. The previous studies have shown the formation of functional anastomoses between murine and human vasculature within the grafts, allowing the preservation of a fully functional human microvascular compartment. This included the expression of disease-specific vascular addressins, pro-inflammatory cytokines, and the preservation of functional lymphoid aggregates with local B-cell differentiation and anti-cyclic citrullinated peptide (anti-CCP) antibody production (10, 15, 41, 42).

The previous data from our group have demonstrated that intra-graft injections of human TNF can stimulate the expression of cellular adhesion molecules (CAM), promoting *in vivo* recruitment of peripheral blood lymphocytes (15). Similarly, TNF blockade should reduce the expression of CAMs and other markers associated with inflammation, resulting in a decreased cellularity and resolution of inflammation. Early studies on the activity of anti-TNF antibodies have demonstrated that treatment efficacy was accompanied by a decrease in synovial cellularity and a reduction of CD68^+^ cells in RA patients (43–45). Treatment of grafted mice with IV injections of the BsAb A7/adalimumab was more effective than adalimumab scFv-Fc in reducing synovial cellularity with a more pronounced effect on CD68^+^ cells. This was mirrored by a reduction of GM-CSF mRNA expression. Further, treatment with A7/adalimumab was also able to efficiently reduce mRNA expression and serum concentrations of pro-inflammatory cytokines, such as IL-8 and TNF, while other cytokines such as IL-1β and IL-6 showed a downregulation, without reaching statistical significance. These results are consistent with the previous reports in the literature, where TNF blockade is associated with the reduction of key pro-inflammatory cytokines, such as IL-1, IL-6, IL-8, and GM-CSF (23, 24, 46). Elevated expression of MMPs in the synovium has been previously described in RA (25–28) and the treatment with anti-TNF agents has shown to downregulate MMP expression in patients (47). Similarly, A7/adalimumab was able to significantly decrease MMP-1 and MMP-3 mRNA expression in the grafts with a more potent effect compared to adalimumab scFv-Fc. Overall, the BsAb A7/adalimumab consistently showed a potent and efficient anti-inflammatory activity within the arthritic synovial grafts.

In summary, here we described a novel therapeutic antibody with improved potency and tissue specificity and provided evidence for increased pharmacological activity in the human arthritic synovium with a strong indication for efficacy in the human disease. By design, the BsAb developed in this study contained only one anti-TNF binding domain. It is plausible to assume that by maintaining an efficient anti-TNF activity in the disease tissue with only half TNF binding potential, compared to standard therapy, would translate in lower systemic TNF engagement and increased safety. In practical terms, the use of such BsAb molecules in the clinical care of chronic arthritis may also allow for a reduced treatment regimen, owing to the improved potency, and consequently reducing the associated healthcare costs. Furthermore, this new BsAb construct has the potential to represent a novel platform for tissue-specific drug delivery in RA by combining the unique properties of the A7 synovial targeting domain to the effector functions of appropriate anti-rheumatic biologics. This strategy could conceivably be extended to other existing anti-cytokine antibodies with the intent of developing therapeutic agents with reduced systemic exposure, improved potency, enhanced safety profile, and reduced treatment costs.

## Data Availability Statement

The original contributions generated for the study are included in the article/Supplementary Material, further inquiries can be directed to the corresponding author/s.

## Ethics Statement

The studies involving human participants were reviewed and approved by East London and The City Research ethics Committee 3, and the NRES Committee London—City and East. Rec No. is 07/Q0605/29. The patients/participants provided their written informed consent to participate in this study. The animal study was reviewed and approved by Local Animal Ethics and Welfare Committee under Home Office regulation.

## Author Contributions

MF designed, performed experiments, analyzed results, and wrote the manuscript. SO designed, performed experiments, analyzed results and reviewed the manuscript. LF-J, AN, FC, SP, and CD performed experiments and analyzed results. PA performed experiments. CS and DS analyzed results and reviewed the manuscript. CP designed experiments, analyzed results, and reviewed the manuscript. All authors contributed to the article and approved the submitted version.

## Conflict of Interest

CP is an author in a patent related to the A7 antibody. The remaining authors declare that the research was conducted in the absence of any commercial or financial relationships that could be construed as a potential conflict of interest.

## Abbreviations

RA: rheumatoid arthritis
BsAb: bispecific antibody
NSAID: non-steroidal anti-inflammatory drug
sDMARD: synthetic disease-modifying antirheumatic drug
bDMARD: biologic disease modifying antirheumatic drug
scFv: single-chain variable fragment
His: histidine
EC_50_: effective concentration 50%
IC_50_: inhibitory concentration 50%
KD: kinetic constant
Ka: association kinetic
Kd: dissociation kinetic
MMP: matrix metallo-proteinase
TIMP: tissue-inhibitor of metallo-proteinase
M-CSF: macrophage colony-stimulating factor
GM-CSF: granulocyte-macrophage colony stimulating factor
CIA: collagen induced arthritis
EDB: extra-domain B of fibronectin
EDA: extradomain A of fibronectin
CCP: cyclic citrullinated peptide
CAM: cellular adhesion molecule
CHO: Chinese hamster ovary
SPR: surface plasmon resonance
DAB: 3,3′-diamino-benzidine
vWF: von Willebrand factor
DSF: differential scanning fluorimetry.
