# Supplementary material for: Novel Bispecific Antibody for Synovial-Specific Target Delivery of Anti-TNF Therapy in Rheumatoid Arthritis
Source: Front Immunol. 2021 Feb 19;12:640070. doi: 10.3389/fimmu.2021.640070 (PMC7933454; doi:10.3389/fimmu.2021.640070)
Supplement: Supplementary file 1 [file Data_Sheet_1.DOCX]

Supplementary Material

# Supplementary Figures and Tables

## Supplementary Figures

**Differential scanning fluorimetry (DSF)**

Purified antibodies were loaded in a glass capillary and thermo stability measured over a range of temperature from 20 to 95 °C with 1 °C increments per minute on a Prometheus NT.48 instrument. Unfolding was determined using 330nm/350nm ratio and first derivative to derive Tm values. All antibodies demonstrated similar Tm value for the first unfolding event, involving the scFv domains, with a 1.4 °C total variation. The presence of the Y407T and T366Y mutations caused a reduced stability for the Fc domain of the bispecific construct.


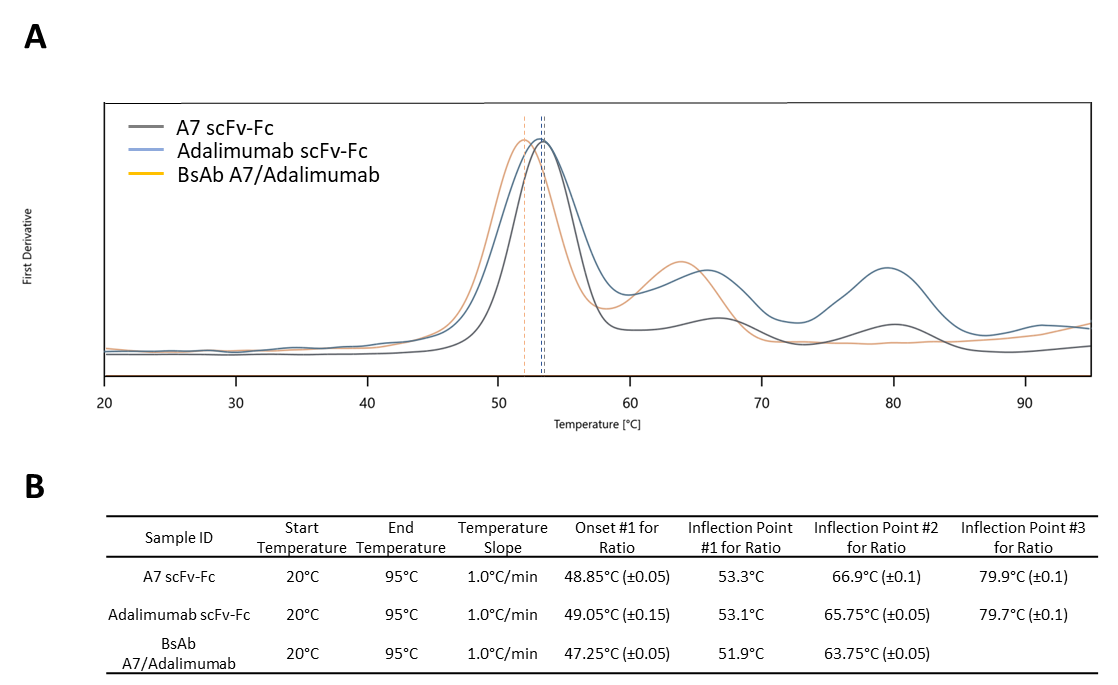


**Supplementary Figure 1**. **A)** First derivative of 330nm/350nm ratio for A7 scFv-Fc (grey), Adalimumab scFv-Fc (blue) and BsAb A7/Adalimumab (orange) showing similar unfolding events. Dotted lines represent calculated first unfolding Tm for each antibody. **B)** Table showing Tm temperatures for unfolding onset, first domain unfolding (scFv) and subsequent unfolding events (Fc).

**Heterodimerization efficiency**

To measure heterodimerization efficiency of the knob-into-hole technology described in the manuscript, a construct was generated removing the scFv domain from the T366Y Fc chain. Full length and truncated chains were expressed in a monocistronic manner and carrying a 2A peptide at the interface to allow for efficient 1:1 expression ratio. Antibodies were expressed and purified as described in the main text. Presence of homodimer and heterodimer conformations were analyzed by SDS-PAGE and identified via difference in molecular weight.


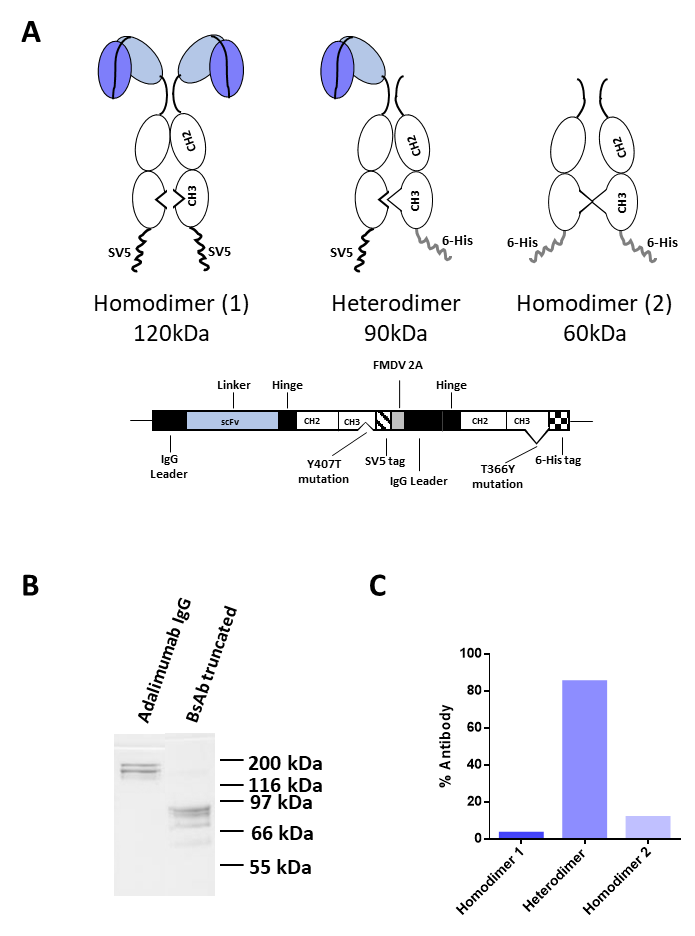


**Supplementary Figure 2.** **A**) A truncated knob-into-hole bispecific antibody construct was design by removing the scFv domain associated to the T366Y Fc chain. The resulting antibody consists in a heterodimer format at 90 kDa and two putative homodimer forms at 60 and 120 kDa. (**B**) SDS-PAGE analysis in non-reducing conditions of the purified antibody from cell culture supernatant using Talon® metal affinity chromatography (Clonetech) shows a predominant band at 90 kDa for the truncated bsAb. (**C**) Densitometry analysis of the SDS-PAGE BsAb lane using the imaging software tool ImageJ 1.44p (NIH, USA) identifies 85% of efficient heterodimerization with 3% and 11% of homodimer forms at 120 kDa and 60 kDa, respectively.

**Comparison of *in vivo* tissue localisation efficiency**


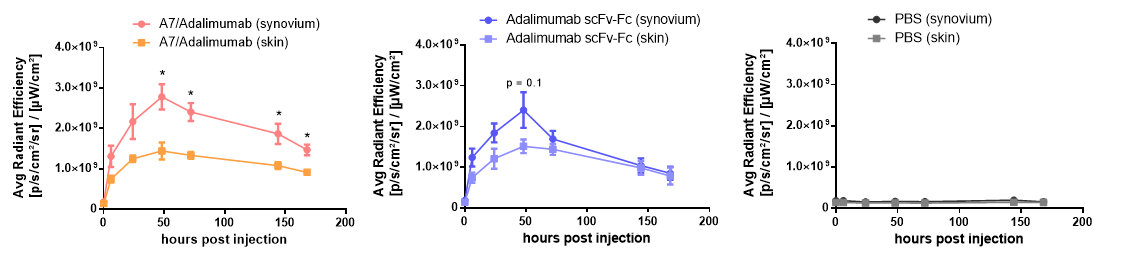


**Supplementary Figure 3.** Comparison of average radiant efficiency in the region of interest encompassing the synovium graft (n=4) and skin graft (n=4) for A7/Adalimumab BsAb, Adalimumab scFv-Fc and PBS i.v. injected SCID mice. In vivo fluorescence measured at 6, 24, 48, 72, 144 and 168 hours post-injection. Mann-Whitney non parametric test (* = p<0.05)

**Serum half-life of injected antibodies**


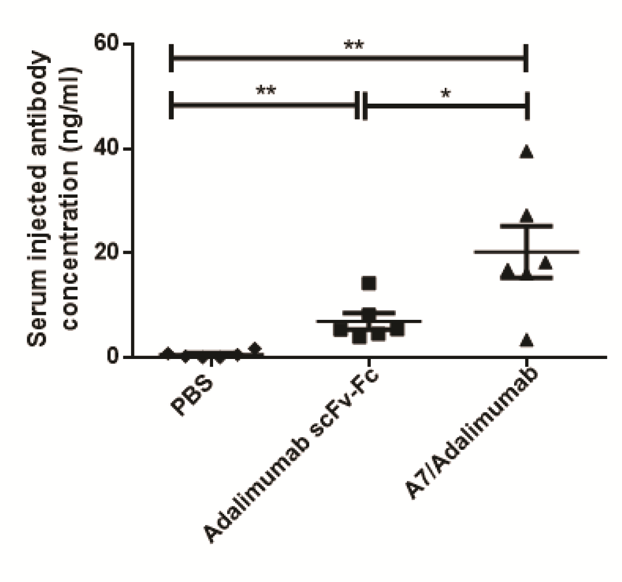


**Supplementary Figure 4.** Concentration of human Fc tagged antibodies in the serum of mice 1 week after IV injection (n=6). A7/Adalimumab BsAb shows significantly higher serum titer over Adalimumab scFv-Fc. Mann-Whitney non parametric test (**= p<0.01, *= p<0.05))
